# Reducing the stigma of mental illness in undergraduate medical education: a randomized controlled trial

**DOI:** 10.1186/1472-6920-13-141

**Published:** 2013-10-24

**Authors:** Andriyka Papish, Aliya Kassam, Geeta Modgill, Gina Vaz, Lauren Zanussi, Scott Patten

**Affiliations:** 1 Department of Psychiatry, College of Medicine - Regina Campus, University of Saskatchewan, 2110 Hamilton St, Regina, SK S4P 2E3, Canada; 2 Department of Community Health Sciences, Faculty of Medicine, University of Calgary, 3330 Hospital Drive NW, Calgary, AB T2N 4N1, Canada; 3 Opening Minds Anti-Stigma Initiative, Mental Health Commission of Canada, 110 Quarry Park Blvd, Suite 320, Calgary, Alberta T2C 3G3, Canada; 4 Department of Psychiatry, Faculty of Medicine, University of Calgary, 1403 - 29 Street NW, Calgary, Alberta T2N 2T9, Canada; 5 Department of Community Health Sciences, University of Calgary, 3rd Floor TRW, 3280 Hospital Drive NW, Calgary T2N 4Z6, Canada

**Keywords:** Stigma, Medical education, Mental illness, Psychiatry, Contact-based education, Knowledge, Process, Randomized controlled trial

## Abstract

**Background:**

The stigma of mental illness among medical students is a prevalent concern that has far reaching negative consequences. Attempts to combat this stigma through educational initiatives have had mixed results. This study examined the impact of a one-time contact-based educational intervention on the stigma of mental illness among medical students and compared this with a multimodal undergraduate psychiatry course at the University of Calgary, Canada that integrates contact-based educational strategies. Attitudes towards mental illness were compared with those towards type 2 diabetes mellitus (T2DM).

**Method:**

A cluster-randomized trial design was used to evaluate the impact of contact-based educational interventions delivered at two points in time. The impact was assessed by collecting data at 4 time points using the Opening Minds Scale for Health Care Providers (OMS-HC) to assess changes in stigma.

**Results:**

Baseline surveys were completed by 62% (n=111) of students before the start of the course and post-intervention ratings were available from 90 of these. Stigma scores for both groups were significantly reduced upon course completion (p < 0.0001), but were not significantly changed following the one-time contact based educational intervention in the primary analysis. Student confidence in working with people with a mental illness and interest in a psychiatric career was increased at the end of the course. Stigma towards mental illness remained greater than for T2DM at all time points.

**Conclusions:**

Psychiatric education can decrease the stigma of mental illness and increase student confidence. However, one-time, contact-based educational interventions require further evaluation in this context. The key components are postulated to be contact, knowledge and attention to process, where attending to the student’s internal experience of working with people with mental illness is an integral factor in modulating perceptions of mental illness and a psychiatric career.

## Background

Mental illness remains profoundly stigmatized despite numerous initiatives to combat the negative stereotypes [1-5]. Many of these campaigns have focused on changing the attitudes of medical professionals, as they often carry an equal or greater degree of stigma towards mental illness than do those in the general public [1,5-11]. Stigma can be understood as a combination of problems of knowledge (ignorance), attitudes (prejudice) and behaviour (discrimination) [3] and has been described as a “primary barrier” to treatment and recovery [1,12]. It can be particularly damaging when it comes from medical professionals, to whom people turn for help, and has well documented detrimental effects on both patient care and physician health [2,7-9,13]. Not only does stigma add to disease burden by preventing people from seeking timely help [14], but it is also perpetuated as future generations of doctors assimilate stereotypes from the medical culture [1,15]. Thus, there is a need to explore effective interventions that reduce the negative attitudes that health care providers may have towards individuals with mental illness.

Amongst health care providers, medical students are a particularly important group to target with regards to attitudes towards people with mental illness. Attitudes early on in training tend to be more amenable to change and tend to harden as students progress through medical school and residency [15-17]. As future doctors, they will be influential in shaping the culture of medicine and their responses, or lack of, to incidents of stigmatizing behavior or attitudes will model for others what physicians consider to be appropriate behavior [1]. Furthermore, medical students and physicians are at higher risk of burnout and addictions than others in the general public yet are reluctant to seek help due to the associated stigma [13,18,19]. Part of this reluctance might also stem from the potential negative consequences on a physician’s career if they disclose having a mental illness, as medicine is a regulated profession in which disclosure of a mental illness can limit a physician’s ability to practice [20]. It has also been postulated that the stigma of mental illness contributes to the shortage of medical students choosing a psychiatric career, due to perceptions of it being an unrewarding and stressful profession [17,21,22]. Students are exposed to a medical culture in which psychiatrists have a more pessimistic view of mental illness than those in the general public [7-9]. This “physician bias” may be due to psychiatrist’s clinical experiences of trying to treat those who are most ill, do not recover fully or relapse frequently, ultimately shaping their perspectives on mental illness, recovery and patient care [10]. Thus, finding effective methods to improve medical student attitudes towards mental illness and psychiatry may be a potent way to disrupt the cycle of stigma in the medical culture and improve patient care [23].

According to a recent position paper by the Canadian Psychiatric Association, “conventional education on mental illness and mental health alone does not help reduce stigmatizing attitudes and behaviors” [1] and some have even described medical schools as a “breeding ground for stigma and discrimination” towards mental illness [1,2,7]. Numerous strategies have been proposed to help combat mental illness stigma in medical students, including knowledge and contact-based initiatives [1,2,5,6,24-27]. Among these, contact-based educational strategies have emerged as particularly effective and involve statements made by people about their lived experience of mental illness and their interaction with the health care system [1,2,25-27]. This strategy of using social contact aims to reduce stigma by providing the opportunity for interpersonal contact between people who have a history of mental illness and audiences who may be stigmatizing towards them [24]. Although some medical schools employ contact-based teaching methods as part of their psychiatric curricula, few have evaluated the effectiveness of the programs on reducing the stigma of mental illness and increasing student confidence in working with people with mental illness.

Furthermore, stigma has been criticized as being too vaguely defined and individually focused [28] and thus existing models have defined stigma as a dynamic interrelationship of components. This interrelationship involves cognitive, affective and behavioural components. In our study we focus on the affective component of stigma.

Our conceptualization of stigma is the tri-partite model, which proposes that stigma is an overarching term including three core elements: knowledge (misinformation/differences in understanding due to culture or religion), attitudes (prejudice) and behaviour (discrimination) [29]. The knowledge, attitudes and behaviour framework allows clear intervention targets and units of measurement [29,30]. The importance of knowledge, attitudes and behaviour has been established in medical education with medical students, nurses, and other health care providers [29-32]. The knowledge, attitudes and behaviour framework is also one that is widely used in health promotion [33,34]. The tri-partite model focuses on the problem of attitudes in the form of prejudice which can be elicited as common stereotypes or emotional reactions rather than separating them like other out-dated models. The tri-partite model is adaptable in that it allows for attitudes towards people with mental illness to be comprised of the various dimensions of stigma [29].

These dimensions include: 'perceived stigma’, which refers to one’s belief that others perceive an individual as socially unacceptable [9,34-36] and 'self-stigma’, which refers to a similar, internalized perception of oneself leading to the fear of seeking help or disclosing one’s mental illness due to the stigma associated with mental illness [37]. Other dimensions of stigma also include social distance, which refers to one’s desire to maintain distance from people with mental illness [28,38], 'dangerousness’, which refers to one’s belief that the individual is dangerous [36], recovery, which refers to one’s belief that people with mental illness can recover [39]. Emotional reactions [40] such as a lack of social responsibility as well as a lack of empathy or compassion towards people with mental illness are also dimensions of stigma [40]. While some may consider that compassion and social responsibility may be paternalistic and stigmatizing towards people with mental illness [41], these dimensions can be seen as important indicators of non-stigmatizing attitudes and are acknowledged widely as significant competencies of health care providers and those in training to become a health care provider [9,42].

In our study we targeted attitudes with our intervention which was contact-based. We conducted a cluster-randomized, “wait-list” controlled trial to evaluate the impact of a one-time contact-based educational intervention on the stigma of mental illness as measured by attitudes among medical students as compared with a multimodal undergraduate psychiatry course that integrates contact-based educational strategies. Attitudes towards mental illness were compared with those towards type 2 diabetes mellitus (T2DM), a stigmatized but non-mental health-related illness [43].

## Methods

A randomized control trial was designed to assess the impact of two different educational interventions on medical student attitudes towards mental illness: a one-time contact based educational intervention and a 4 week mandatory psychiatry course at the University of Calgary, in Calgary (U of C), Canada. The Psychiatry and Family Violence Course is part of the U of C Medical School’s three-year, year-round program where clinical presentations are the foundation of the curriculum [44] and the majority of students have an undergraduate or graduate university degree prior to entering medical school. Students completed the course in their second year immediately prior to starting the clerkship component of their education.

The course learning objectives were based on the Medical Council of Canada’s (MCC) Objectives for the Qualifying Examination Part I, which are based on the Royal College of Physicians and Surgeons of Canada’s CanMEDS (Canadian Medical Educations Directives for Specialists) framework for physicians’ roles [45].

The course content was organized according to the following topics: Psychiatric interviewing and the Mental Status Examination, addictions, ADHD, anxiety disorders, eating disorders, emergency psychiatry, psychiatry and the law, psychiatry of the elderly, organic causes of mental illnesses, mood disorders, mood disorders in children and adolescents, personality disorders, pharmacology, psychosis, psychosomatic disorders, psychotherapy and the general physician, the sexually concerned patient, suicide, family issues and violence.

Students were taught about mental illness using the biopsychosocial model [46]. Mental disorders were classified according to criteria established by the Diagnostic and Statistical Manual of Mental Disorders (DSM-IV-TR) [47].

The course has been the highest rated course in the medical school based on student evaluations for the last seven years, and incorporates various teaching methods including didactic teaching (30 hours), case-based teaching with group discussions (12 hours) and an optional movie night with a post-movie discussion about mental illness (3–4 hours). It also includes two teaching methods that involved contact with people who have mental illness: patient presentations (2 hours) and “clinical correlations” sessions (6 hours).

“Clinical correlations” are small group teaching sessions where 5–6 students, paired since the first day of medical school, are mentored by a psychiatrist and directly interact with patients with a mental illness in an inpatient or out-patient setting. They provide students with an opportunity to practice their psychiatric skills and to process their reactions and experiences with the psychiatrist. Anecdotal reports suggest that students value these sessions (6 hours in total) and we hypothesized that students would rank this course component as the most effective teaching method.

The patient presentation component (a one-time contact based educational intervention) consisted of two, one-hour oral presentations given by patients who shared their story of having a mental illness. The first patient had recovered from medication-induced depression with psychotic symptoms and the second patient had narcolepsy and narcissistic personality disorder. Students had an opportunity to ask questions of the presenters and of the psychiatry course chair, who moderated the sessions.

To determine the impact of the entire psychiatry course on the stigma of mental illness and how this compared with the one-time contact-based educational intervention, students were cluster randomized according their clinical correlation groups into either an early intervention group or the late intervention group using a computer generated random sequence. The early group (intervention group) received the patient presentation on the first day of class, prior to the commencement of the regular course curriculum. The late intervention group (control group) received the same presentation at the very end of the course. Apart from the timing of the contact-based sessions, both groups participated in the same curriculum.

Data were collected on-line using the Opening Minds Scale for Health Care Providers (OMS-HC; Additional file 1), a validated twenty item scale [48]. Data were collected at four different time points: prior to the beginning of the course (T1), after a randomization step on the first day of class (T2), upon completion of the course (T3), and three months after the course was completed (T4). To assess for the consistency of student responses over time and to place medical student attitudes towards mental illness into a greater context, attitudes towards mental illness were compared with those for Type 2 Diabetes Mellitus (T2DM), a stigmatized but non-mental health related illness [43], using questions 4, 5, 6 and 7 of the OMS-HC (Additional file 1). The third survey, upon course completion, included additional questions to assess medical student perceptions of the course and its impact on their attitudes and behaviors.

The study was approved by the U of C Ethics Board and participation was voluntary. Students were assigned a unique identifier to maintain confidentiality and investigators directly involved with the course remained blinded to student participation. The following demographic information was collected to assess its impact on medical student attitudes: age, gender, contact with people with mental illness (family member, close friend, or having treated a patient with mental illness) and a personal history of being treated for a mental illness. Students were also asked if they have completed any psychiatry electives and if they would consider a career in psychiatry.

Data was entered twice and checked for errors and outliers. The analysis used the statistical program STATA version 11.0 [49]. Authors used frequency distributions to characterize the participants with respect to their responses on each individual questionnaire item. Two-way frequency distributions and cross-tabulations were used in the analysis.

Attitudes towards people with mental illness were measured at baseline (T1) and compared to the ratings after the intervention (T2) and at the end of the course (T3). Attitudes were found to be substantially right skewed, whereas changes in those ratings were approximately normally distributed. The authors therefore examined changes between the various sets of ratings, so that parametric statistical tests could be used. The primary analysis examined the change between the first and second set of ratings to allow for a comparison of changes at a point in time where one half of the sample had received the contact-based intervention (intervention group) and one had not (control group). It was restricted to people who completed the baseline (T1) and post-intervention survey (T2), and linear regression analysis with adjustment for baseline score was used in these comparisons. The null hypothesis that the change score did not depend on group was assessed using a likelihood ratio test. The unit of randomization (clinical correlation groups) was included in the analysis as strata. The three month follow-up provided an opportunity to examine whether changes observed during the course were sustained. The secondary analysis examined the change between the baseline ratings and the post-course ratings.

## Results

### Sample characteristics

Of the 179 students eligible to participate in the study, 111 completed a baseline survey (62.0% response rate). Of these, 81.0% (n=90) completed the second survey, 86.5% (n=96) completed the third survey and 52.1% (n=50) completed the 3 month follow-up survey. Although 96.1% (n=172) of the class responded to the third survey, only data from students who completed the baseline survey was used to assess the impact of the contact-based interventions.

### Baseline measures

Participant characteristics are summarised in Table 1. Approximately two-thirds of respondents who completed the baseline survey were female (59.5%), between the ages of 18–25 (63.9%), and of “white” ethnicity (69.9%). Over 88% of students did not have any previous exposure to psychiatry through electives and 13.5% reported that they had been treated for a mental illness in the past. The second and third columns of Table 1 compare participant characteristics across the intervention and control groups. The two groups differed by age and interest in a psychiatric career, with more older students and those with a greater baseline interest in psychiatry in the control group. There were no other statistically significant differences between the two groups.

**Table 1:** Description of study sample at baseline (n = 111), n (%)

	**Study sample (n = 111) n, (%)**	**Intervention group (n = 55) n, (%)**	**Control group (n= 56) n, (%)**	**P-value**
Gender				
Male	45 (40.5)	18 (32.7)	27 (48.2)	0.07^a^
Female	66 (59.5)	37 (67.3)	29 (51.8)	
Age				
18–25 years	71 (63.9)	41 (74.5)	30 (53.8)	0.02^b^
26–44 years	40 (36.1)	14 (25.5)	26 (46.4)	
Ethnicity				
White	76 (69.9)	38 (69.1)	38 (67.9)	0.50^a^
Asian	16 (14.4)	8 (14.6)	8 (14.3)	
South East Asian	16 (14.4)	6 (10.9)	10 (17.9)	
Other	3 (2.7)	3 (5.5)	-	
Summer Psychiatry Elective				
No	99 (89.2)	51 (92.8)	48 (85.7)	0.20^a^
Yes	12 (10.8)	4 (7.3)	8 (14.3)	
Fall/Winter Psychiatry Elective				
No	98 (88.3)	51 (92.8)	48 (85.7)	0.29^a^
Yes	13 (11.7)	4 (7.3)	8 (14.3)	
Have treated a patient for a mental illness				
No	57	26 (47.3)	31 (55.4)	
Yes	46	25 (45.5)	21 (37.5)	0.67^a^
Don’t Know	8	4 (7.3)	4 (7.1)	
Have been treated for a mental illness				
No	96 (86.5)	47 (84.5)	49 (87.5)	0.49^a^
Yes	15 (13.5)	8 (14.6)	7 (12.5)	
Would consider a career in Psychiatry				
No	53 (47.8)	30 (54.6)	23 (41.1)	
Yes	25 (22.5)	5 (9.1)	20 (35.7)	0.003^a^
Maybe	33 (29.7)	20 (36.4)	12 (23.2)	

^a^Fisher’s exact test, ^b^Mann–Whitney test.

### OMS-HC scores

The internal consistency of the OMS-HC, measured using Cronbach’s alpha was 0.78 at baseline, 0.75 at T2, 0.81 at T3, and 0.84 at T4. The OMS-HC produces scores between 20 (no stigma) and 100 (extreme stigma). The overall OMS-HC totals at each of the four time points were T1 = 48.6 (47.5-49.8); T2 = 48.1 (47.1-49.2); T3= 44.2 (42.8 - 45.4) and T4 = 45.2 (43.1-47.3). Table 2 shows unadjusted total stigma scores over time stratified according to intervention group. Negative change scores demonstrate decreases in stigma. At each of the four time points, there was no statistically significant difference in the scores between the two groups. Scores were lowest (least stigma) immediately following the psychiatry course.

**Table 2:** OM survey totals, by group

	**Sample size (*n*)**	**Intervention group**		**Control group**			
		**n**	**Total score (95% CI)**	**n**	**Total score (95% CI)**	**Difference**	**p-value**
T1	111	55	49.3 (47.5 – 51.2)	56	47.9 (46.4 – 49.4)	1.4	0.29
T2	90	48	49.0 (47.7 – 50.3)	42	47.2 (45.4 – 48.9)	1.8	0.18
T3	96	45	45.1 (43.1 – 47.1)	51	43.3 (41.6 – 48.9)	1.8	0.23
T4	53	26	45.7 (42.6 – 48.8)	27	44.7 (41.5 – 47.8)	1.0	0.66

T1 = baseline; T2 = following the intervention; T3 = following the course; T4 = 3 month follow-up. A higher score correlates with greater stigma.

### Impact of the one-time contact based intervention

The primary analysis examined if the changes in OMS-HC score depended on group. The analysis was restricted to students (n=90) that completed the baseline (T1) and post-intervention (T2) surveys. Table 3 shows the change scores between different time points for all participants and for the two groups. The model included adjustment for baseline OMS-HC scores. The null hypothesis that the change score did not depend on group was assessed via likelihood ratio tests for the main effect of group. This test showed no evidence that the change between T1 and T2 differed by group (p=0.05). There was no statistical evidence that the one-time contact-based educational intervention had differing effects on men and women.

**Table 3:** Changes in unadjusted OMS-HC scores according to intervention group, by study interval

	**T2-T1 n = 90**	**T3-T1 n=96**	**T3-T2 n=96**	**T4-T1 n= 50**	**T4-T3 n=50**
All participants	-0.4 (-1.0, 0.3) p = 0.374	-4.4 (-5.8, -3.1)^*^ p<0.0001	-4.2 (-5.6, -2.9)^*^ p<0.0001	-3.4 (-5.9, -1.9)^*^ p = 0.0002	1.0 (-0.2, 2.2) p = 0.10
Intervention group	0.3 (-0.5, 1.0) p = 0.67	-4.2 (-6.4, -2.0)^*^ p<0.0001	-4.6 (-6.6, -2.7)^*^ p<0.0001	-3.6 (-6.5, -0.8)^*^ p = 0.0148	1.5 (-0.3, 3.3) p = 0.06
Control group	-1.0 (-2.2, 0.1) p = 0.05	-4.6 (-6.4, -3.0)^*^ p<0.0001	-3.9 (-5.7, -2.0)^*^ p=0.0002	-3.2 (-7.1, -1.3)^*^ p = 0.006	0.5 (-1.3, 2.3) p = 0.58

T1 = baseline; T2 = following the intervention; T3 = following the course; T4 = 3 month follow-up. Negative scores denote a decrease in stigma; change score (95% CI), p-value.

*= Statistically significant, based on one-sample t-tests (where H_o_: change=0 and α = 0.05).

However, as there were statistically significant differences between the two groups despite randomisation (Table 1) a model was created to adjust for baseline score, age, sex, career intention and group (Table 4, Model 1). After this adjustment there was a statistically significant difference in the change scores between the two groups between T1 and T2 (p = 0.03), suggesting that the effect of contact was significant. The model was repeated using a command to adjust for clustering based on clinical correlations groups (Table 4, Model 2) which again suggested that the one-time contact based educational intervention had a significant effect (p = 0.01). However, it should be emphasized that these were not preplanned analyses.

**Table 4:** Post-Hoc analysis using linear regression models for OMS-HCP change score by group, gender, age, and interest in psychiatry

	**Model 1**	**Model 2**
	**Coefficient (95% CI)**	**Coefficient (95% CI)**
Intervention group	-1.7 (-3.2 to -0.2)^a^	-1.7 (-2.8 to -0.5)^b^
Baseline OMS-HCP score	-0.2 (-0.3 to -0.1)^a^	-0.2 (-0.3 to -0.1)^c^
Female	-1.4 (-3.0 to 0.1)	-1.4 (-3.2 to 0.3)
Age 18 to 25	0.6 (-0.2 to 0.4)	0.6 (-0.7 to 2.0)
Would consider a career in Psychiatry	0.1 (-0.2 to 0.4)	0.1 (-0.2 to 0.3)

Model 1: Model does not adjust for clustering, Model 2: Model adjusts for clustering of clinical correlation groups (using STATA [49]: svy command); SE = standard error.

^a^p value <0.05.

^b^p value <0.01.

^c^p value <0.001.

### Impact of the course

The secondary analysis examined the impact of the psychiatry course on the overall OMS-HC scores. The analysis included respondents who had completed the baseline (T1) and post-course surveys (T3). Negative scores correlate with less stigma. The psychiatry course was associated with a significant overall decrease in change scores: -4.4 OMS-HC points (95% CI: -5.8 to -3.1; p<0.0001), suggestive of less stigma upon course completion. An analysis of differences between T2 (start of the course but after one group received an intervention) and the end of the course (T3) also showed a significant overall decrease in change scores for both groups. An exploratory analysis found that covariates (age, sex, previous mental health electives, receiving treatment for a mental illness, treating a patient with a mental illness) did not predict post-course stigma scores. Stigma ratings at the 3-month follow-up remained significantly lower than baseline ratings and were not significantly different from scores at the end of the course.

### Attitudes and disclosure/help-seeking sub-scales

Additional analysis examined the impact of the course on two sub-scales [29]: 1) attitudes towards people with mental illness and 2) disclosure and help seeking (Additional file 1). The psychiatry course was associated with a significant overall decrease (improvement) in both attitude items [-1.6 OMS-HC points (95% CI: -2.2 to -1.0; p<0.0001)] and disclosure items [-2.3 OMS-HC points (95% CI: -2.9 to -1.7; p<0.0001)].

### Attitudes towards mental illness vs type 2 diabetes mellitus

**Figure 1:**
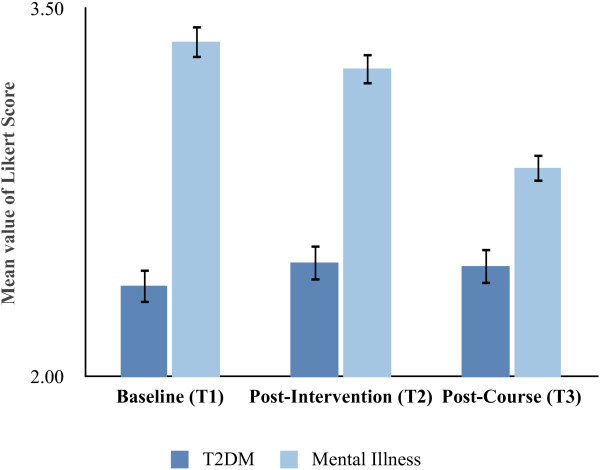
**Mean Likert scores for medical student responses to four questions about attitudes towards mental illness and type 2 diabetes mellitus (T2DM), asked at three different time points.** A larger value corresponds with more stigmatizing attitudes. Error bars indicate standard error. T1 = baseline; T2 = following the intervention; T3 = following the course.

Student survey responses were placed into a greater context by comparing their attitudes towards mental illness with T2DM. Figure 1 shows the combined mean Likert scores for four questions comparing mental illness stigma with T2DM stigma at three different times. At all three time point students carried more stigma towards mental illness than towards T2DM. No significant differences were observed between the control and intervention groups and there was no significant difference in medical student attitudes towards T2DM at each of the time points. With regards to attitudes towards mental illness, there was no difference in mean responses towards mental illness at T1 vs T2 but there was a significant improvement in student attitudes towards mental illness upon completion of the course (T3) when compared with baseline (T1) and with T2. Despite this improvement, student attitudes towards mental illness continued to be more stigmatised than those for T2DM upon course completion.

### Interest in psychiatry

There was an increased interest in psychiatry as a career upon completion of the course. Of those that replied to the first three surveys, 25 students indicated that “yes” they would consider a career in psychiatry prior to the start of the course. This decreased to 13 after the first patient presentation and increased to 28 upon course completion.

### Evaluation of the course and course components

Additional questions were asked at the end of the course (T3) to assess medical student perceptions of the course and its impact on their attitudes and behaviors (n = 174). The vast majority of students (89%, CI: 82.9-95.1%) thought that the course was “useful in reducing prejudice and discrimination against people with mental illness” and two thirds (66%, 95% CI: 56.8%-75.2%) thought that their “behavior towards people with mental illness will be different than what it would have been before Course VII”. The two contact-based educational methods emerged with the highest rankings when students ranked the effectiveness of each teaching method for learning about people with mental illness: 49% ranked the clinical correlations as “extremely effective” compared with 26 % for the patient presentations, clinical cases (18%), didactic teaching (4%) and movie night (3%). The students also ranked the clinical correlations component as having the greatest impact on increasing their confidence in working with people with mental illness: 58% strongly agreed vs 40% for didactic teaching and 33% for patient presentation (additional data not shown).

## Discussion

This study is novel in its use of a cluster randomized control trial to evaluate contact-based educational interventions aimed at reducing the stigma of mental illness among medical students. The results support previous findings which show that medical students do carry a stigma towards mental illness [17,19]. It also supports the notion that comprehensive medical education can be effective in reducing the stigma of mental illness and can increase medical students’ confidence in working with people with mental illness. These results suggest that it is possible to create an environment in which medical student attitudes towards mental illness can be shifted in a positive direction. However, despite the improvement in attitudes, the stigma towards mental illness remained greater than that for a stigmatized physical illness [43], type 2 diabetes mellitus.

In contrast to the positive impact of the course, the evidence was less robust that one-time contact-based educational sessions can alter medical student attitudes towards mental illness as an effect was only evident in post-hoc analysis. Although contact-based education has been proposed as a key component in stigma reduction strategies [1,24,50,51] contact alone may be insufficient to significantly alter medical student attitudes towards mental illness. Some have suggested that the type of contact needs to be appropriate, where, for example, patients must be seen as having equal status [4] and where the person has successfully recovered from a mental illness [1]. Thus perhaps the patient presentations in this study were not adequate in disconfirming stereotypes of people with mental illness or the study was underpowered.

However, such requirements may not be realistic representations of clinical practice. Medical students and physicians often work with individuals with severe and refractory illness, thus see a skewed sample of those with mental illness [7,8]. Furthermore, there is an innate power differential that exists between a physician and a patient [25,52,53] which may contribute to stigma and to the notion of “us” and “them” [1].

Although the evidence for one-time contact based sessions altering stigma was less robust, the course as a whole was clearly effective in improving students’ attitudes towards mental illness. One possibility is that these medical students required additional knowledge, acquired throughout the course, to help tackle the stigma of mental illness. Accurate knowledge can help correct misinformation, reduce ignorance and improve mental health literacy, especially when combined with contact strategies [6,25,51]. However, some types of knowledge might not be helpful in reducing the stigma of mental illness. There is emerging evidence that education about the biological nature of mental illness and framing it as a neurological brain disease may perpetuate the stigma by suggesting to some that it is irreversible and creating wider social divisions [1,54,55].

Moreover, the combination of knowledge and contact has not consistently reduced stigma in medical students [2,14,23]. Researchers have been further perplexed that improvements in attitudes towards mental illness do not necessarily translate into an increased interest in pursuing a psychiatric career [24], suggesting underlying concerns remain. This may be related to the stress that medical students experience when treating very ill or challenging patients on psychiatric rotations [21,22,56]. Furthermore, students can struggle with under empathizing or over-empathizing, potentially leading to poor quality care and burn out among medical students and physicians [22,56]. These factors likely contribute to medical students lacking the confidence that they can help those with mental illness [22]. Student attitudes towards mental illness are also influenced by the culture of the medical community, which has historically had negative views of psychiatry and psychiatrists [2,8,57]. This includes both physician and the allied mental health care providers [2,8]. Thus, although students may have improved attitudes towards psychiatry and mental illness, they may be reluctant to pursue a career in which they themselves are stigmatized “by association” [2,7,57]. Ultimately, these factors might not only result in fewer medical students choosing a psychiatric career, but could also impact the treatment that people with mental illness receive in all areas of medicine [23].

**Figure 2:**
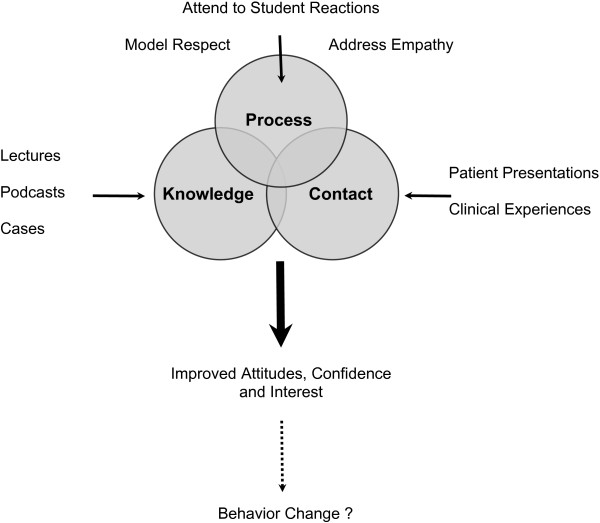
**Model for decreasing stigma and improving medical student attitudes towards mental illness.** The authors propose that changing medical student attitudes towards mental illness requires a combination of accurate knowledge, contact-based educational methods and attention to process factors. In this model, attending to the student’s internal experience of working with people with mental illness is particularly important as it provides an opportunity to correct misconceptions that have occurred as a result of student’s knowledge and contact-based educational experiences, and can help increase student confidence in working with people with mental illness.

Perhaps there is an additional component that is integral in shaping medical student attitudes - attention to process (Figure 2). Process refers to “how” we do things and non-verbal behavior [58]. Recently the Canadian Psychiatric Association [1] advocated for psychiatrists to act as role models for trainees by directly addressing, debriefing and processing incidents of stigma and discrimination, and suggested that this can be a powerful tool in combating stigma and discrimination. Others have also found that “face-to-face” clinical teaching is highly valued by students and contributes to positive attitudes towards psychiatry [59], and conclusions drawn from a recent meta-analysis suggest that there is a need to “identify moderators of the effects of both education and contact” [5].

When examining the effectiveness of the U of C course’s individual components, the clinical correlations sessions were rated as most effective in increasing student confidence in working with people with mental illness. In this particular component of the course, attention is paid to the student’s experience of working with people at various stages of recovery from mental illness. Medical students have an opportunity to discuss their internal reactions with a psychiatrist, who can help them make sense of these. It also creates an opportunity for psychiatrists to correct misconceptions that may have arisen as a result of medical students’ education (knowledge) and clinical (contact) experiences. One could therefore speculate that paying attention to process is a crucial step in not only reducing stigma but in increasing future physician’s confidence in treating people with mental illness.

Thus, perhaps the course’s success in improving attitudes towards mental illness is due to the combined approach of knowledge, contact and attention to process: it provides a solid grounding in knowledge through the use of diverse teaching strategies that include contact-based educational methods, and perhaps most importantly, attention is paid to the student’s experience of working with people with mental illness.

One limitation of this study is that we were unable to assess the specific impact of the clinical correlations sessions due to methodological limitations such as each group having their sessions at different time points throughout the course. Future studies using qualitative approaches to examine this aspect of the course in greater depth could help ascertain which factors within the clinical correlations sessions are of greatest benefit in reducing stigma and improving student interest in psychiatry. The study may also have been underpowered to detect differences based on age, gender, past exposure to psychiatry and personal history of mental illness. Surprisingly, the student response rate was greater after the course (96%) than at baseline (62%) which could be indicative of greater student engagement following the course. This may potentially be a source of bias given that only those who initially responded were included when comparing stigma scores.

Our 3 month follow-up data had a reduced response rate and students had varied clinical experiences during the follow-up time frame depending upon which clerkship rotations they had completed. Thus, caution needs to be taken when interpreting this data. However, the availability of national statistics for this class’ career choices provides some helpful information about student’s ultimate first choice of residency greater than one year later: 9.7% (n=17) of the U of C medical students ultimately chose and matched to psychiatry as their first choice of career, a higher percentage than any other Canadian medical school and almost twice as high as the national 2012 average for Canadian Medical Graduates (5.1%) (CaRMS) [60] and US medical graduates (3.9%) [61]. This suggests that this cohort’s interest in psychiatry persisted beyond the course and throughout clerkship. A future follow-up study of these same students while in residency would be helpful to measure how exposure to the medical culture impacts medical student attitudes towards mental illness and if changes in attitudes correspond with knowledge and behavioral changes. For example, oral exams can be used to investigate knowledge and objective structured clinical examinations (OSCEs) can be used to examine behavior. This would take into account all components of the tri-partite model [29].

## Conclusions

The stigma of mental illness among medical students is a prevalent concern that has far reaching negative consequences, yet not all educational interventions have been effective in reducing this stigma. Reducing the stigma of mental illness appears to require the combined effect of various components within medical education curricula: knowledge, contact-based interventions, and attending to the student’s internal experience of working with people with mental illness.

## Abbreviations

T2DM: Type 2 diabetes mellitus; OMS-HC: Opening minds scale for health care providers; U of C: University of Calgary; T1: Time point 1, prior to the beginning of the course; T2: Time point 2, after half the class receives the intervention on the first day of class; T3: Time point 3, upon completion of the course; T4: Time point 4, three months after the course was completed; CI: Confidence interval; CaRMS: Canadian residency matching service.

## Competing interests

The authors declare that they have no competing interests.

## Authors’ contributions

AP designed and conducted the study, was involved in data interpretation and drafted the manuscript. AK assisted with study design and oversaw data collection and data analysis. GM was involved with implementing the study, data collection and statistical analysis. GV assisted with implementation of the study. LZ was the course chair and assisted with study design, patient coordination and supervision of AP. SP provided guidance throughout all stages of the study and supervision of AK and GM. All authors were involved with writing and editing the manuscript. All authors read and approved the final manuscript.

## Authors’ information

At the time this study was conducted, AP was a psychiatry resident in the University of Calgary’s Faculty of Medicine and is now a clinical assistant professor at the University of Saskatchewan’s College of Medicine, Regina Campus. AK was a research associate with the Opening Minds Initiative for the Mental Health Commission of Canada and is now an assistant professor in the Department of Community Health Sciences within the University of Calgary’s Faculty of Medicine. GM is a research associate with the Mental Health Commission of Canada. GV was a senior medical student at the University of Calgary Medical School at the time of data collection. LZ was the chair of the undergraduate psychiatry course and is a clinical assistant professor in the Department of Psychiatry within the University of Calgary’s Faculty of Medicine. SP was the post-doctoral supervisor for AK, is a professor in the Departments of Community Health Sciences and Psychiatry at the University of Calgary and a Senior Health Scholar with Alberta Innovates, Health Solutions.

## Pre-publication history

The pre-publication history for this paper can be accessed here:

http://www.biomedcentral.com/1472-6920/13/141/prepub

## Supplementary Material

Additional file 1 OMS-HC scale and associated type 2 diabetes mellitus questions. Click here for file

## References

[B1] Abbey S Charbonneau M Tranulis C Moss P Baici W Dabby L Gautam M Paré M Stigma and discrimination [position paper] Can J Psychiatry 2011 56 10 1 9 22014688

[B2] Sartorius N Gaebel W Cleveland HR Stuart H Akiyama T Arboleda-Florez J Baumann AE Gureje O Jorge MR Kastrup M Suzuki Y Tasman A WPA guidance on how to combat stigmatization of psychiatry and psychiatrists World Psychiatry 2010 9 3 131 144 20975855 10.1002/j.2051-5545.2010.tb00296.x PMC2948719

[B3] Thornicroft G Rose D Kassam A Sartorius N Stigma: ignorance, prejudice or discrimination? Br J Psychiatry 2007 190 192 193 10.1192/bjp.bp.106.025791 17329736

[B4] Corrigan PW Penn DL Lessons from social psychology on discrediting psychiatric stigma Am Psychol 1999 54 9 765 776 10510666 10.1037//0003-066x.54.9.765

[B5] Corrigan PW Morris SB Michaels PJ Rafacz JD Rusch N Challenging the public stigma of mental illness: a meta-analysis of outcome studies Psychiatr Serv 2012 63 10 963 973 10.1176/appi.ps.201100529 23032675

[B6] Thornicroft G Brohan E Kassam A Lewis-Holmes E Reducing stigma and discrimination: candidate interventions Int J Ment Health Syst 2008 2 1 3 10.1186/1752-4458-2-3 18405393 PMC2365928

[B7] Thornicroft G Rose D Mehta N Discrimination against people with mental illness: what can psychiatrists do? Adv Psychiatr Treat 2010 16 53 59 10.1192/apt.bp.107.004481

[B8] Schultz B Stigma and mental health professionals: a review of the evidence on an intricate relationship Int Rev Psychiatry 2007 19 2 137 155 10.1080/09540260701278929 17464792

[B9] Corrigan P How stigma interferes with mental health care Am Psychol 2004 59 7 614 625 15491256 10.1037/0003-066X.59.7.614

[B10] Jorm AF Korten AE Jacomb PA Christensen H Henderson S Attitudes towards people with a mental disorder: a survey of the Australian public and health professionals Aust N Z J Psychiatry 1999 33 1 77 83 10.1046/j.1440-1614.1999.00513.x 10197888

[B11] Nordt C Rossler W Lauber C Attitudes of mental health professionals towards people with schizophrenia and major depression Acta Psychiatr Scand 2006 114 2 145 146 16836605 10.1111/j.1600-0447.2006.00864.x

[B12] US Dept of Health and Human Services Mental health: a report of the surgeon general 1999 Bethesda, MD: US Department of Health and Human Services

[B13] Gautam M Goldman LS, Myers M, Dickstein LJ Physicians and Depression The Handbook of physician health: the essential guide to understanding the health needs of physicians 2000 Chicago, IL: American Medical Association 80 94

[B14] Smith JK Weaver DB Capturing medical students’ idealism Ann Fam Med 2006 4 S32 S37 10.1370/afm.543 17003160 PMC1578670

[B15] Nieuwhof MG Rademakers JJ Kuyvenhoven MM Soethout MB Ten Cate TJ Students’ conceptions of the medical profession: an interview study Med Teach 2008 27 709 714 16451892 10.1080/01421590500271159

[B16] Korszun A Dinos S Ahmed K Kamaldeep B Medical attitudes about mental illness: does medical-school education reduce stigma? Acad Psychiatry 2012 36 197 204 22751821 10.1176/appi.ap.10110159

[B17] Schwenk TL Davis L Wimsatt LA Depression, stigma, and suicidal ideation in medical students JAMA 2010 304 11 1181 1190 10.1001/jama.2010.1300 20841531

[B18] Dyrbye LN Thomas MR Massie FS Power DV Eacker A Harper W Durning S Moutier C Szydio DW Novotny PJ Sloan JA Shanafelt TD Burnout and suicidal ideation among U.S. medical students Ann Intern Med 2008 149 5 334 341 10.7326/0003-4819-149-5-200809020-00008 18765703

[B19] Chew-Graham CA Rogers A Yassin N 'I wouldn’t want it on my CV or their records’: medical students’ experiences of help-seeking for mental health problems Med Educ 2003 37 10 873 880 10.1046/j.1365-2923.2003.01627.x 12974841

[B20] Canadian Medical Protective Association (CMPA) Physician personal Health Information: Supporting public safety and individual privacy 2010 http://www.cmpa-acpm.ca/cmpapd04/docs/submissions_papers/com_physician_personal_health_information-e.pdf

[B21] Cutler JL Harding KJ Mozian SA Wright LL Pica AG Masters SR Graham MJ Discrediting the notion “working with 'crazies’ will make you 'crazy”’: addressing stigma and enhancing empathy in medical student education Adv in Health Sci Educ 2009 14 487 502 10.1007/s10459-008-9132-4 18766453

[B22] Cutler JL Alspector SL Harding KJ Wright LA Graham MJ Medical students’ perceptions of psychiatry as a career choice Acad Psychiatry 2006 30 144 149 10.1176/appi.ap.30.2.144 16609121

[B23] Feldman TB Medical students’ attitudes toward psychiatry and mental disorders Acad Psychiatry 2005 29 4 4 356 10.1176/appi.ap.29.4.354 16223897

[B24] Pinfold V Thornicroft G Huxley P Farmer P Active ingredients in anti-stigma programmes in mental health Int Rev Psychiatry 2005 17 2 123 131 10.1080/09540260500073638 16194782

[B25] Arboleda-Florez J Stuart H From sin to science: fighting the stigmatization of mental illnesses Can J Psychiatry 2012 57 8 457 463 22854027 10.1177/070674371205700803

[B26] Couture S Penn DL Interpersonal contact and the stigma of mental illness: a review of the literature J Ment Health 2003 12 3 291 303 10.1080/09638231000118276

[B27] Kassam A Glozier N Leese M Loughran J Thornicroft G A controlled trial of mental illness related stigma training for medical students BMC Med Educ 2011 11 1 1 51 10.1186/1472-6920-11-1 21801355 PMC3161004

[B28] Link BG Cullen FT Frank J Wozniak J The social rejection of ex-mental patients: understanding why labels matter Am J Sociol 1987 92 1461 1500 10.1086/228672

[B29] Thornicroft G Shunned: Discrimination against people with mental illness 2006 New York: Oxford University Press

[B30] Johnston JM Leung GM Fielding R Tin KY Ho LM The development and validation of a knowledge, attitude and behaviour questionnaire to assess undergraduate evidence-based practice teaching and learning Med Educ 2003 37 992 1000 10.1046/j.1365-2923.2003.01678.x 14629412

[B31] Hoke MM Robbins LK Continuing the cultural competency journey through exploration of knowledge, attitudes, and skills with advanced practice psychiatric nursing students: an exemplar Nurs Clin North Am 2011 46 2 201 205 10.1016/j.cnur.2011.02.004 21501731

[B32] Kajermo KN Tyni-Lenné R Guidetti S Samuelsson M Andersson IL Wengström Y Evidence-based practice: attitudes, knowledge and behaviour among allied health care professionals Int J Qual Health Care 2011 23 2 198 209 10.1093/intqhc/mzq083 21242158

[B33] La Torre G Semyonov L Mannocci A Boccia A Knowledge, attitude, and behaviour of public health doctors towards pandemic influenza compared to the general population in Italy Scand J Public Health 2011 0 1 7 10.1177/1403494811424612 22006167

[B34] National Institute for Health and Clinical Excellence Behaviour change at population, community and individual levels 2007 London, England: National Institute for Health and Clinical Excellence

[B35] Link BG Cullen FT Contact with the mentally ill and perceptions of how dangerous they are J Health Soc Behav 1986 27 289 302 10.2307/2136945 3559124

[B36] Corrigan P Markowitz FE Watson A Rowan D Kubiak MA An attribution model of public discrimination towards persons with mental illness J Health Soc Behav 2003 44 162 179 10.2307/1519806 12866388

[B37] Corrigan PW Watson AC The paradox of self-stigma and mental illness Clin Psychol Sci Pract 2002 9 1 35 53 10.1093/clipsy.9.1.35

[B38] Bentz WK Hollister WG Kherlopian M Attitudes of social distance and social responsibility for mental illness: a comparison of teachers and the general public Psychol Sch 1970 7 2 198 203 10.1002/1520-6807(197004)7:2<198::AID-PITS2310070218>3.0.CO;2-D

[B39] Mueser KT Corrigan PW Hilton DW Illness management and recovery: a review of the research Psychiatr Serv 2002 53 1272 1284 10.1176/appi.ps.53.10.1272 12364675

[B40] Angermeyer MC Holzinger A Matschinger H Emotional reactions to people with mental illness Epidemiol Psichiatr Soc 2010 19 1 26 32 20486421 10.1017/s1121189x00001573

[B41] Jacoby A Felt versus enacted stigma: a concept revisited: Evidence from a study of people with epilepsy in remission Soc Sci Med 1994 38 2 269 274 10.1016/0277-9536(94)90396-4 8140453

[B42] Spandler H Stickley T No hope without compassion: the importance of compassion in recovery-focused mental health services J Ment Health 2011 20 6 555 566 10.3109/09638237.2011.583949 22126632

[B43] Hopper S Diabetes as a stigmatized condition: the case of low-income clinic patients in the United States Soc Sci Med Med Anthropol 1981 15B 1 11 19 7209601 10.1016/0160-7987(81)90004-1

[B44] University of Calgary Medical School operating Philosophy 2010 Calgary, Canada http://www.ucalgary.ca/mdprogram/operatingphilosophy

[B45] Medical Council of Canada’s (MCC) Objectives for the Qualifying Examination Part Ihttp://mcc.ca/examinations/objectives-overview/

[B46] Engel GL The clinical application of the biopsychosocial model Am J Psychiatry 1980 137 535 544 7369396 10.1176/ajp.137.5.535

[B47] American Psychiatric Association Diagnostic and statistical manual of mental disorders 2000 4 Washington, DC: American Psychiatric Association

[B48] Kassam A Papish A Modgill G Patten S The development and psychometric properties of a new scale to measure mental illness related stigma by health care providers: the opening minds scale for health care providers (OMS-HC) BMC Psychiatry 2012 12 62 10.1186/1471-244X-12-62 22694771 PMC3681304

[B49] Stata Corporation Stata, version 11.0. [11.0] 2009 College Station, TX: Stata Corporation

[B50] Nguyen E Chen TF O’Reilly CL Evaluating the impact of direct and indirect contact on the mental health stigma of pharmacy students Soc Psychiatry Psychiatr Epidemiol 2012 47 7 1087 1098 10.1007/s00127-011-0413-5 21755345

[B51] Corrigan PW River LP Lundin RK Penn DL Uphoff-Wasowski K Campion J Mathisen J Gagnon C Bergman M Goldstein H Kubiak MA Three strategies for changing attributions about severe mental illness Schizophr Bull 2001 27 2 187 195 10.1093/oxfordjournals.schbul.a006865 11354586

[B52] Nadelson C Noteman M Boundaries in the doctor-patient relationship Theor Med Bioeth 2002 23 3 191 201 10.1023/A:1020899425668 12467344

[B53] Link BG Phelan JC Conceptualizing stigma Annu Rev Sociol 2001 27 363 385 10.1146/annurev.soc.27.1.363

[B54] Schnittker J An uncertain revolution: why the rise of a genetic model of mental illness has not increased tolerance Soc Sci Med 2008 67 9 1370 1381 10.1016/j.socscimed.2008.07.007 18703264

[B55] Pescosolido B Martin J Long JS Medina T Phelan J Link B “A disease like any other?” a decade of change in public reactions to schizophrenia, depression, and alcohol dependence Am J Psychiatry 2010 167 1321 1330 10.1176/appi.ajp.2010.09121743 20843872 PMC4429867

[B56] Niedermier JA Bornstein R Brandemihl A The junior medical student psychiatry clerkship: curriculum, attitudes, and test performance Acad Psychiatry 2006 30 136 143 10.1176/appi.ap.30.2.136 16609120

[B57] Kurtz S Silverman J Benson J Draper J Marrying content and process in clinical method teaching: enhancing the calgary-cambridge guides Acad Med 2003 78 802 809 10.1097/00001888-200308000-00011 12915371

[B58] Lampe L Coulston C Walter G Mahli G Up close and personal: medical students prefer face-to-face teaching in psychiatry Australas Psychiatry 2010 18 4 354 360 10.3109/10398561003739620 20645903

[B59] Canadian Residency Matching Service (CaRMS) Reports and Statistics 2012 http://www.carms.ca/eng/operations_R1reports_12_e.shtml

[B60] Mark M Fewer US Grads matching in psychiatry Am Psychiatr Assoc Psychiatr News 2012 47 8 1b 30b

[B61] Stampfer H The recruitment problem in psychiatry: a critical commentary Educ Res Perspect 2011 38 2 1 19

