# Supplementary material for: Reducing the stigma of mental illness in undergraduate medical education: a randomized controlled trial
Source: BMC Med Educ. 2013 Oct 24;13:141. doi: 10.1186/1472-6920-13-141 (PMC3828029; doi:10.1186/1472-6920-13-141)
Supplement: Additional file 1 — OMS-HC scale and associated type 2 diabetes mellitus questions. [file 1472-6920-13-141-S1.doc]

Additional file 1: OMS-HC Scale and associated Type 2 Diabetes Mellitus Questions.

The Attitude toward people with mental illness sub-scale [29] consists of questions 1, 2, 12, 13, 14, 18, 20. The Disclosure / Help seeking sub-scale consists of questions 4, 5, 6, 7, 10. Most questions have a negative orientation such that higher ratings mean more stigma. Seven items have a positive orientation: 3,8,9,10,11,15 and 19. Replies were based on a 5-point Likert scale (strongly disagree, disagree, neutral, agree, strongly agree), where negatively oriented questions were scored 1, 2, 3, 4 or 5 and the positively oriented questions were scored 5, 4, 3, 2, 1.

1. I am more comfortable helping a person who has a physical illness than I am helping a person who has a mental illness.

2. If a person with a mental illness complains of physical symptoms (e.g. nausea, back pain or headache), I would likely attribute this to their mental illness.

3. If a colleague with whom I work told me they had a managed mental illness, I would be as willing to work with him/her.

4. If I were under treatment for a mental illness I would not disclose this to any of my colleagues.

5. I would be more inclined to seek help for a mental illness if my treating healthcare provider was not associated with my workplace.

6. I would see myself as weak if I had a mental illness and could not fix it myself.

7. I would be reluctant to seek help if I had a mental illness.

8. Employers should hire a person with a managed mental illness if he/she is the best person for the job.

9. I would still go to a physician if I knew that the physician had been treated for a mental illness.

10. If I had a mental illness, I would tell my friends.

11. It is the responsibility of health care providers to inspire hope in people with mental illness.

12. Despite my professional beliefs, I have negative reactions towards people who have mental illness.

13. There is little I can do to help people with mental illness.

14. More than half of people with mental illness don’t try hard enough to get better.

15. People with mental illness seldom pose a risk to the public.

16. The best treatment for mental illness is medication.

17. I would not want a person with a mental illness, even if it were appropriately managed, to work with children.

18. Healthcare providers do not need to be advocates for people with mental illness.

19. I would not mind if a person with a mental illness lived next door to me.

20. I struggle to feel compassion for a person with a mental illness.

Type 2 Diabetes Mellitus questions:

1. If I were under treatment for Type 2 Diabetes Mellitus I would not disclose this to any of my colleagues.

2. I would be more inclined to seek help for Type 2 Diabetes Mellitus s if my treating healthcare provider was not associated with my workplace.

3. I would see myself as weak if I had a Type 2 Diabetes Mellitus and could not fix it myself.

4. I would be reluctant to seek help if I had Type 2 Diabetes Mellitus.
